# Exploring molecular signatures related to the mechanism of aging in different brain regions by integrated bioinformatics

**DOI:** 10.3389/fnmol.2023.1133106

**Published:** 2023-03-23

**Authors:** Xie Su, Lu Xie, Jing Li, Xinyue Tian, Bing Lin, Menghua Chen

**Affiliations:** ^1^Department of Intensive Care Unit, The Second Affiliated Hospital of Guangxi Medical University, Nanning, China; ^2^Department of Physiology, Pre-Clinical Science, Guangxi Medical University, Nanning, China

**Keywords:** brain aging, brain regions, molecular signatures, neuroinflammation, bioinformatics, hub genes

## Abstract

The mechanism of brain aging is not fully understood. Few studies have attempted to identify molecular changes using bioinformatics at the subregional level in the aging brain. This study aimed to identify the molecular signatures and key genes involved in aging, depending on the brain region. Differentially expressed genes (DEGs) associated with aging of the cerebral cortex (CX), hippocampus (HC), and cerebellum (CB) were identified based on five datasets from the Gene Expression Omnibus (GEO). The molecular signatures of aging were explored using functional and pathway analyses. Hub genes of each brain region were determined by protein–protein interaction network analysis, and commonly expressed DEGs (co-DEGs) were also found. Gene–microRNAs (miRNAs) and gene–disease interactions were constructed using online databases. The expression levels and regional specificity of the hub genes and co-DEGs were validated using animal experiments. In total, 32, 293, and 141 DEGs were identified in aging CX, HC, and CB, respectively. Enrichment analysis indicated molecular changes related to leukocyte invasion, abnormal neurotransmission, and impaired neurogenesis due to inflammation as the major signatures of the CX, HC, and CB. Itgax is a hub gene of cortical aging. Zfp51 and Zfp62 were identified as hub genes involved in hippocampal aging. Itgax and Cxcl10 were identified as hub genes involved in cerebellar aging. S100a8 was the only co-DEG in all three regions. In addition, a series of molecular changes associated with inflammation was observed in all three brain regions. Several miRNAs interact with hub genes and S100a8. The change in gene levels was further validated in an animal experiment. Only the upregulation of Zfp51 and Zfp62 was restricted to the HC. The molecular signatures of aging exhibit regional differences in the brain and seem to be closely related to neuroinflammation. Itgax, Zfp51, Zfp62, Cxcl10, and S100a8 may be key genes and potential targets for the prevention of brain aging.

## Introduction

As an inevitable physiological phenomenon, brain aging leads to cognitive declines, including memory, learning ability, attention and processing speed, and neurodegenerative diseases, such as Alzheimer’s disease (AD), dementia, and Parkinson’s disease. These changes contribute to poor quality of life in elderly people and place a heavy burden on society. Currently, there are no effective interventions for delaying the progression of brain aging. One of the important reasons for this dilemma is that the mechanism of brain aging remains unclear, despite tremendous efforts in recent years.

Mechanisms of brain aging generally include mitochondrial dysfunction, impaired DNA repair, aberrant neuronal network activity, stem cell exhaustion, glial cell activation and inflammation, and dysregulated neuronal calcium homeostasis (Yankner et al., 2008; Mattson and Arumugam, 2018). These mechanisms do not usually occur in isolation during the aging process and are highly interdependent and interactive. Therefore, these are common features of aging. However, the aging mechanism may be relatively independent and distinctive in specific brain regions because these regions differ in the proportion of nerve cell subtype and cellular communication. Each region has a specific biological function (Wyss-Coray, 2016). Magnetic resonance imaging has demonstrated that different brain regions did not age at the same pace (Jang et al., 2016; Marjańska et al., 2017). Regional differences in the rate of atrophy of brain tissues during the aging process have been described (Raz et al., 2010). Several biological changes in aging also have regional differences, such as DNA repair capacity, reactive oxygen species levels, metabolome atlas, lipofuscin deposition, and microglial phenotype (Oenzil et al., 1994; Strosznajder et al., 2000; Grabert et al., 2016; Stefanatos and Sanz, 2018; Ding et al., 2021). Therefore, we hypothesize that different brain regions may have different molecular signatures of aging. Exploring the key genes depending on the brain region instead of the whole brain may identify potential key genes of brain aging that may be productively targeted.

In this context, we used a bioinformatics analysis approach to explore molecular alterations in different brain regions. Given that the cerebral cortex (CX), hippocampus (HC), and cerebellum (CB) are associated with cognitive function (Bartsch and Wulff, 2015; Liang and Carlson, 2020; Upright and Baxter, 2021), we selected five datasets that include these three brain regions from the Gene Expression Omnibus (GEO) database for analysis. Differentially expressed genes (DEGs) between young and aged CX, HC, and CB groups were obtained. The DEGs were subjected to functional and pathway analyses to determine the molecular signatures of aging and subjected to R software (RGui) and online analysis to identify the hub genes. The commonly expressed DEGs (co-DEGs) of all three regions were identified to explore the basic and common molecular changes in the aging brain. Subsequently, we constructed the gene–microRNA (miRNA) interactions to explore the potential miRNAs involved in brain aging. Finally, real-time quantitative PCR (RT-qPCR) was used to validate the expression levels and regional specificity of the hub genes and co-DEGs. Overall, this study aimed to identify the molecular signatures and potential key genes involved in brain aging depending on the brain region. This knowledge may enhance the understanding of brain aging at a molecular level.

## Materials and methods

### Data processing

GSE75047, GSE34378, GSE48911, GSE62385, and GSE87102 were downloaded from the GEO database,^1^ a public functional genomics data repository. In GSE75047 (platform GPL10787), samples of the CX and cerebellum CB from 2-month-old and 29-month-old wild-type mice were used for further analysis. Other samples were excluded. Similarly, in GSE34378 (platform GPL14996), CX samples from 3-month-old and 18-month-old mice were selected. In GSE48911 (platform GPL1261), samples of the HC from 4.5-month-old and 20-month-old wild-type mice were selected. In GSE62385 (platform GPL1261), HC samples from 1-month-old and 12-month-old mice from the control group were selected. In GSE87102 (platform GPL7202), samples of CB from 2 to 3-month-old and 21 to 23-month-old mice were selected. We reextracted the gene expression profiles from these datasets as described earlier. In total, 26 young brains and 27 aged brains from mice in these datasets were used for further analysis (Table 1). The expression matrices of the genes were downloaded and normalized using the BiocManager package (version 3.16.0) in the R software (version 4.2.1). After normalization, Sangerbox,^2^ a comprehensive and interaction-friendly clinical bioinformatics analysis platform, was used to merge the matrices of these datasets depending on the brain tissue type and batch differences were removed using the ComBat method (Zhang et al., 2020).

**Table 1:** Samples used in datasets.

Selected samples and ages	Datasets	Strain	Sample preparation	Sequencing method	Tissue type	Sample size
GSM1941427-1941430 (2Mo), GSM1941431-1941434 (29Mo)	GSE75047	C57Bl/6	untreated	GeneChip sequencing (GPL10787)	cortex	8
GSM847849-847851 (3Mo), GSM847858-847860 (18Mo)	GSE34378	C57BL/6 J	untreated	GeneChIP sequencing (GPL14996)	cortex	6
GSM1186705-1186707 (4.5Mo), GSM1186723-1186725 (20Mo)	GSE48911	C57Bl/6	untreated	GeneChIP sequencing (GPL1261)	hippocampus	6
GSM1526451-1526453 (1Mo), GSM1526448-1526450 (12Mo),	GSE62385	C57BL/6 J	untreated	GeneChIP sequencing (GPL1261)	hippocampus	6
GSM1941447-1941450 (2Mo), GSM1941451-1941454 (29Mo)	GSE75047	C57Bl/6	untreated	GeneChip sequencing (GPL10787)	cerebellum	8
GSM2322323-2322327 (2-3Mo), GSM2322338-2322341 (2-3Mo), GSM2322334-2322337 (21-23Mo), GSM2322344-2322349 (21-23Mo)	GSE87102	C57Bl/6 and BALB	untreated	GeneChip sequencing (GPL7202)	cerebellum	19

### Identification of DEGs

DEGs between young (≤4.5 months old) and aged (≥12 months old) groups of CX, HC, and CB were identified by comparing the gene expression of the aged group to that of the young group. The Limma package (version 3.46.0) was used to identify DEGs for each brain region (Ritchie et al., 2015). The Benjamini–Hochberg method was used to adjust the false discovery rate. Genes with a Log2 fold change (FC) > 1 and an adjusted *p*-value of <0.05 were identified as DEGs. DEGs of each brain region were then visualized with a volcano plot created using the ggplot2 package in R software (RGui). A heatmap of DEGs was generated using the Pheatmap package in RGui. The co-DEGs of all three brain regions were also identified by taking the intersection of each set of DEGs using an online tool^3^ and were visualized with a Venn diagram.

### Gene ontology and Kyoto encyclopedia of genes and genomes pathway enrichment analyses

Gene ontology (GO) and Kyoto encyclopedia of genes and genomes (KEGG) enrichment analyses were performed for each set of DEGs using the clusterProfiler package (version 4.6.0) from the Bioconductor platform (Yu et al., 2012). The results were visualized using a bubble chart generated by RGui. The items in the bubble chart of GO enrichment were divided into three categories: biological processes (BP), cellular components (CC), and molecular functions (MF). Statistical significance was set at a *p*-value of <0.05. We performed GO and KEGG enrichment analyses using Metascape,^4^ a powerful online tool for gene function annotation analysis (Xu et al., 2022). The criteria for the analysis were set as follows: minimum overlap = 3, *p*-value cut-off <0.05, and minimum enrichment = 1.5. To confirm the enrichment results, GO and KEGG analyses for DEGs were also performed using the Database for Annotation, Visualization, and Integrated Discovery (DAVID)^5^ online bioinformatics analytical tool for public use, with the criterion of the *p*-value <0.05 (Dennis et al., 2003).

### Protein–protein interaction network construction and identification of hub genes

Protein–protein interaction (PPI) networks of each set of DEGs were established by searching using STRING (version 11.5),^6^ a public data resource used for network functional enrichment analysis of known and predicted PPI (Xu et al., 2022). The minimum required interaction score for network construction was set at <0.4 for the network construction. The disconnected nodes in the network were hidden. Then, RGui and Cytoscape (version 3.8.2)^7^ were used for network analysis, network visualization, and hub gene identification (Xu et al., 2022). When RGui was applied, the hub genes were determined based on the number of gene connections in the networks. We applied Cytohubba in Cytoscape software to calculate the degree of genes using the maximal clique centrality method. Only the genes with the highest (or parallel highest) number of gene connections or degrees were considered hub genes. The results are visualized using bar plots and network diagrams.

### Construction of potential gene–miRNA interactions

We used four online miRNA prediction tools, including six prediction methods. The four prediction tools used were miRBD,^8^ TargetScan (version 8.0),^9^ miRWALK(v 3.0),^10^ and DIANA (including the microT-CDS, microT v4, and TarBase v7.0).^11^ We used these tools to predict the potential miRNAs that could interact with hub genes and co-DEGs (S100a8). We identified three top alternative miRNAs based on the predictive scores for at least two prediction tools for each hub gene and co-DEG. The results are presented in the form of a table.

### Exploring the relationships between aged-related nervous system disease and hub genes/co-DEGs

The Comparative Toxicogenomics Database (CTD)^12^ is a robust, publicly available database that provides manually curated information about chemical–gene/protein interactions and gene–disease relationships (Davis et al., 2021). We used CTD to show the relationship between hub genes/co-DEGs (S100a8) and aging-related nervous system diseases, including neurodegenerative and cerebrovascular diseases.

### Animal treatment

In total, 16 young mice (3 months old) and 16 aged mice (18 months old) were used for experimental validation. C57BL/6 J mice were purchased from Changsha TianQin Biotechnology Technology Corporation Ltd. (Hunan China; animal license No: SCXK (Hunan) 2019-0014). The mice were housed in the Animal Experimental Center of Guangxi Medical University. The mice were housed under environmentally controlled standard indoor conditions (12/12 h light/dark cycle; room temperature, 21°C–24°C; humidity, 50%–70%) with *ad libitum* access to water and food. Mice were anesthetized *via* intraperitoneal injection of pentobarbital (50 mg/kg) and sacrificed by cervical dislocation. A total of 32 samples of CX, HC, and CB were obtained after rapid decapitation of half of the young and aged mice groups. All samples were used to validate gene expressions. Among these samples, seven young and seven aged samples of CX, HC, and CB were used to identify the regional specificity for each hub gene and co-DEG (S100a8). The animal procedures were approved by the Animal Care Welfare Committee of Guangxi Medical University.

### RT-qPCR

The expression level of hub genes and co-DEGs was measured by RT-qPCR. Total RNA was extracted from CX, HC, and CB using the TRIzol reagent (Thermo Fisher Scientific, United States) according to the manufacturer’s instructions. The mRNA was reverse transcribed to cDNA using the Revert Aid First Strand cDNA Synthesis kit (Thermo Fisher Scientific). PowerUp SYBR Green Premix (Thermo Fisher Scientific) was used for qPCR analysis. The cycling conditions were 50°C for 2 min and 95°C for 2 min in the holding stage followed by 40 cycles of 95°C for 15 s and 60°C for 1 min in the cycling stage. Gene expression was normalized to GADPH expression. Relative changes in mRNA levels among groups were determined with the 2^−△△Ct^ method. The primer sequences are listed in Table 2.

**Table 2:** Primers used in qPCR.

Primers	Forward (5′-3′)	Reverse (5′-3′)
Itgax	AGCAGAGCCAGAACTTCCCA	ACTGATGCTACCCGAGCCAT
Zfp51	TCTCATGCAACCAACAGCCT	ACAGTGAGGCTTGAGGAGGT
Zfp62	TGCAGACTCTCAGTGCCCAA	AGTCACAAGTGTCACCGGTCTT
Cxcl10	TGCCGTCATTTTCTGCCTCATCC	TCCCTATGGCCCTCATTCTCACTG
S100a8	TCACCATGCCCTCTACAAGAATGAC	CCATCGCAAGGAACTCCTCGAAG
GADPH	GGTTGTCTCCTGCGACTTCA	TGGTCCAGGGTTTCTTACTCC

### Statistical analyses

Statistical analysis was performed using IBM SPSS Statistics 26. Results were analyzed using an unpaired *t*-test (for normally distributed samples) or a Wilcoxon rank test (for non-normally distributed samples). In the RT-qPCR experiment, more than three wells were added to each sample. Results are expressed as the mean ± SEM. Statistical significance was set at a *p*-value of <0.05.

## Results

### Identification of DEGs

**Figure 1:**
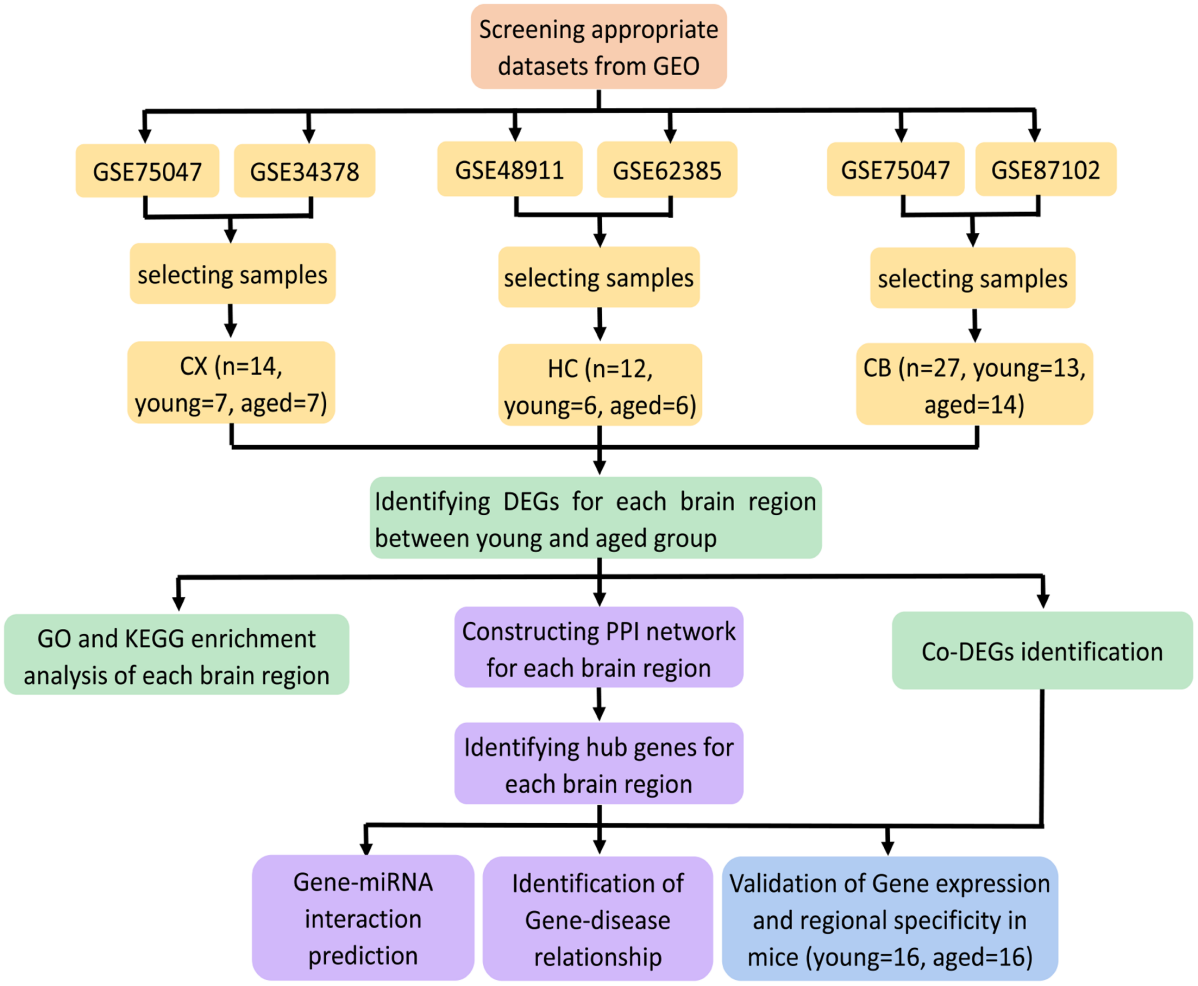
Flowchart of the study.

**Figure 2:**
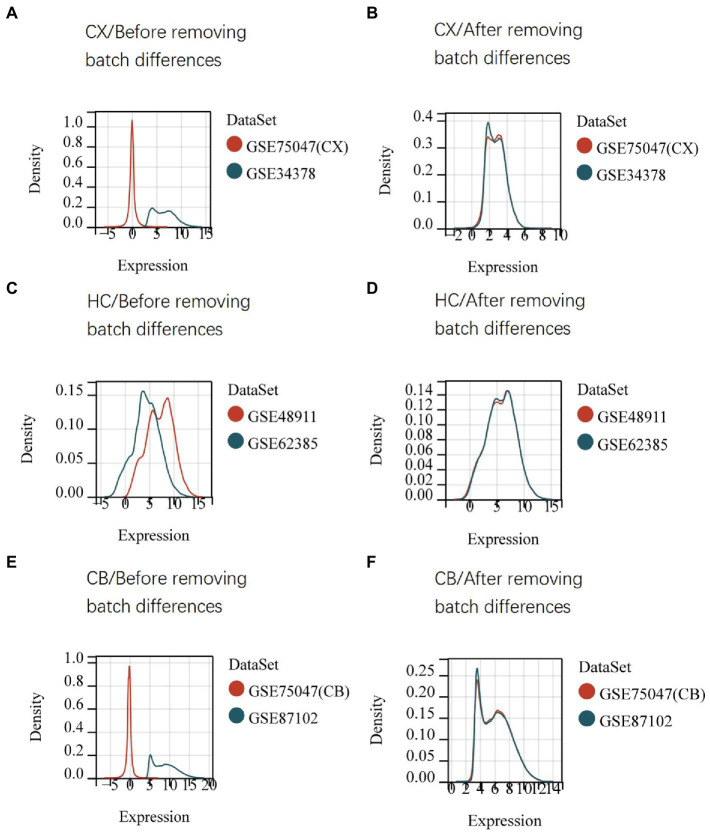
Density distribution map of gene expression in datasets before **(A,C,E)** and after **(B,D,F)** removing batch differences. The maps were generated by SangerBox using the ComBat method. The horizontal axis represents gene expression level and the vertical axis represents probability density. The higher curve overlap, the smaller batch differences. CX, cerebral cortex; HC, hippocampus; CB, cerebellum.

**Figure 3:**
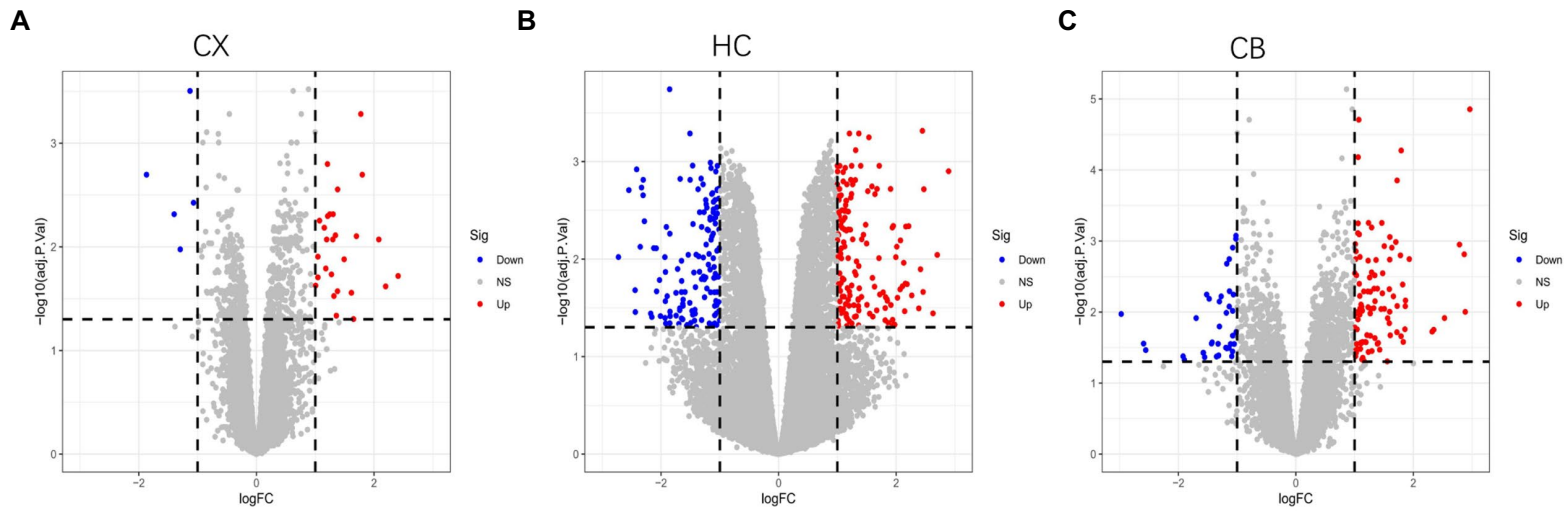
Volcano plots of DEGs between young and aged group in CX **(A)**, HC **(B)** and CB **(C)**. The Limma package was used to identify DEGs. Sample size: CX (Young: *n* = 7, Aged: *n* = 7); HC (Young: *n* = 6, Aged: *n* = 6); CB (Young: *n* = 13, Aged: *n* = 14). Red, blue, and gray nodes represent upregulated genes, downregulated genes and no significantly changed genes, respectively. The genes with |log FC| > 1 and adjusted *p* < 0.05 were identified as DEGs. CX, cerebral cortex; HC, hippocampus; CB, cerebellum.

**Figure 4:**
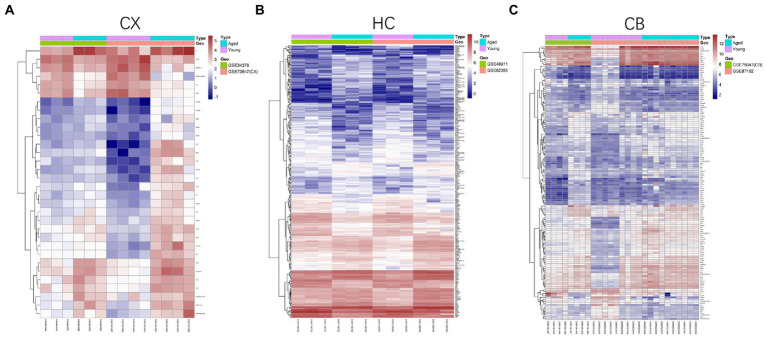
Heatmap of DEGs between young and aged groups of CX **(A)**, HC **(B)** and CB **(C)**. The heatmaps were generated using the Pheatmap package. The horizontal axis represents sample symbol and the vertical axis represents gene symbol. The depth of red (high value) and blue (low value) colors represent height of gene expression value. CX, cerebral cortex; HC, hippocampus; CB, cerebellum.

**Figure 5:**
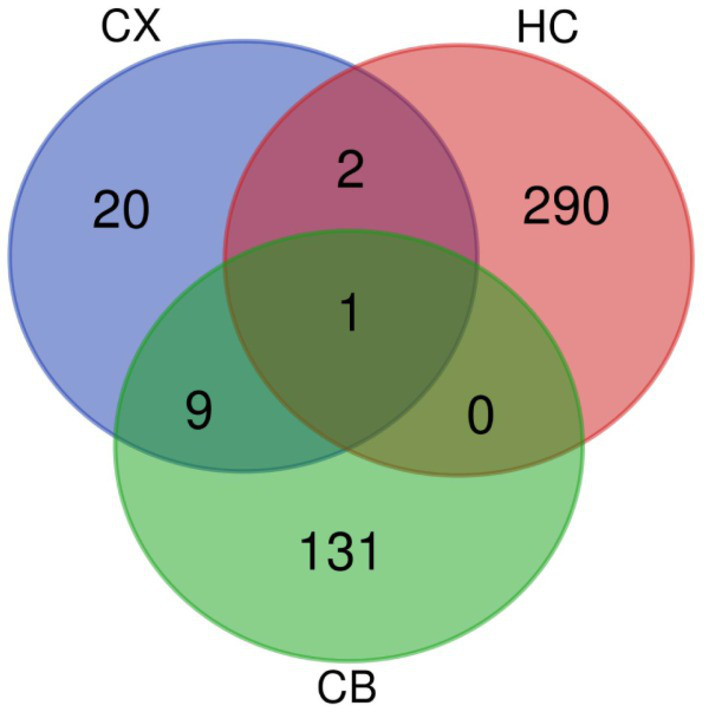
Venn diagram of DEGs. The intersections represent the co-DEGs between each brain region. CX, cerebral cortex; HC, hippocampus; CB, cerebellum.

The procedures for this study are shown in Figure 1. After normalizing the matrices and removing the batch differences, the density distributions of gene expression for each dataset were fundamentally the same. The findings indicate that the data sources were reliable and can be used for further analysis (Figure 2). We identified 32 DEGs (27 upregulated genes and 5 downregulated genes), 293 DEGs (156 upregulated genes and 137 downregulated genes), and 141 DEGs (101 upregulated genes and 40 downregulated genes) between the young and aged CX, HC, and CB groups, respectively. The expression and distribution of DEGs are shown in volcano plots and heatmaps (Figures 3, 4). Co-DEGs responsible for brain aging were also identified. After taking the intersection of the three sets of DEGs, we found 10 co-DEGs between CX and CB, three between CX and HC, and one between HC and CB. S100a8 was the only co-DEG of all three brain regions and was upregulated in the DEGs (Figure 5).

### Go and KEGG enrichment analyses of DEGs

**Figure 6:**
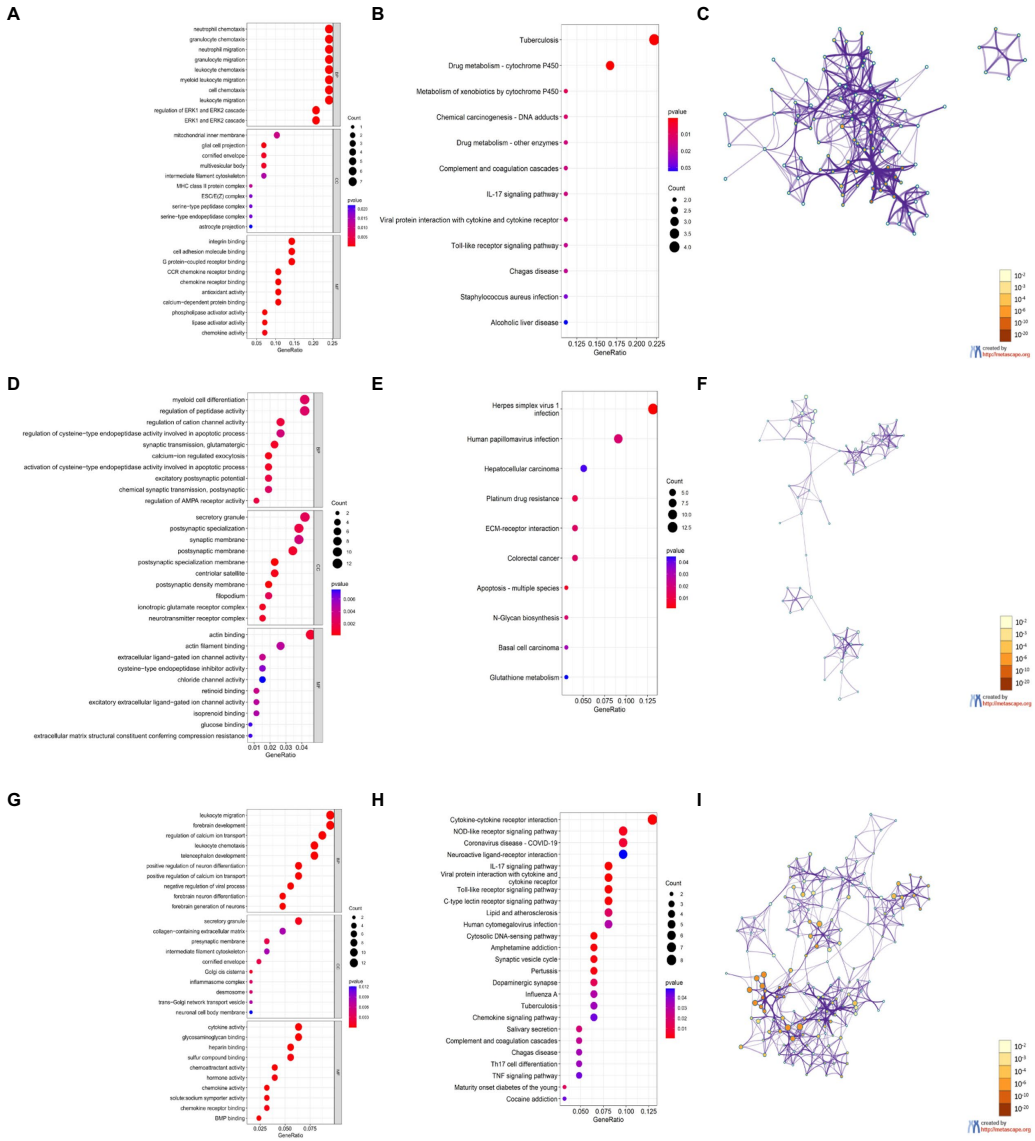
Enrichment analysis of DEGs based on R software and Metascape. **(A,D,G)** GO enrichment items of CX **(A)**, HC **(D)**, and CB **(G)**. **(B,E,H)** KEGG enrichment items of CX **(B)**, HC **(E)**, and CB **(H)**. **(C,F,I)** The main Networks of enrich terms of CX **(C)**, HC **(F)**, and CB **(I)**. **A,B,D,E,G,H** were generated by RGui using the clusterProfiler package. **C,F,I** were generated by Metascape. Statistical significance of enrichment item was set at *p* < 0.05.

The GO and KEGG enrichment results are shown in bubble plots (Figure 6). The GO terms for cortical aging were mainly neutrophil chemotaxis, granulocyte chemotaxis, neutrophil migration, granulocyte migration in BP, glial cell projection, cornified envelope, multivesicular body in CC, integrin binding, cell adhesion molecule binding, G protein-coupled receptor binding, and monocyte C-C motif chemokine receptor (CCR) chemokine receptor binding in MF (Figure 6A). The KEGG pathways of cortical aging included tuberculosis, complement and coagulation cascades, the interleukin (IL)-17 signaling pathway, and the Toll-like receptor signaling pathway (Figure 6B). The GO terms of hippocampal aging were mainly synaptic transmission, calcium ion regulation, α-amino-3-hydroxy-5-methyl-4-isoxazolepropionic acid (AMPA) receptor activity, myeloid cell differentiation, apoptotic process in BP, secretory granule, synaptic, and postsynaptic membrane in CC, and actin binding and actin filament binding in MF (Figure 6D). KEGG pathways of hippocampal aging included apoptosis (Figure 6E). GO terms of cerebellar aging were mainly leukocyte migration, leukocyte chemotaxis, neuron development, neuron differentiation in BP, secretory granule, presynaptic membrane in CC, cytokine activity, chemoattractant activity, chemokine activity, and hormone activity in MF (Figure 6G). The KEGG pathways of cerebellar aging included a series of inflammation-related signaling pathways (Figure 6H). The main parts of the networks of the enriched terms based on Metascape are shown in Figures 6C,F,I. We also applied DAVID to validate the enrichment analysis. The results are provided in the Supplementary material. These enrichment items indicate molecular signatures of aging in different brain regions.

### PPI network analysis and identification of hub genes

**Figure 7:**
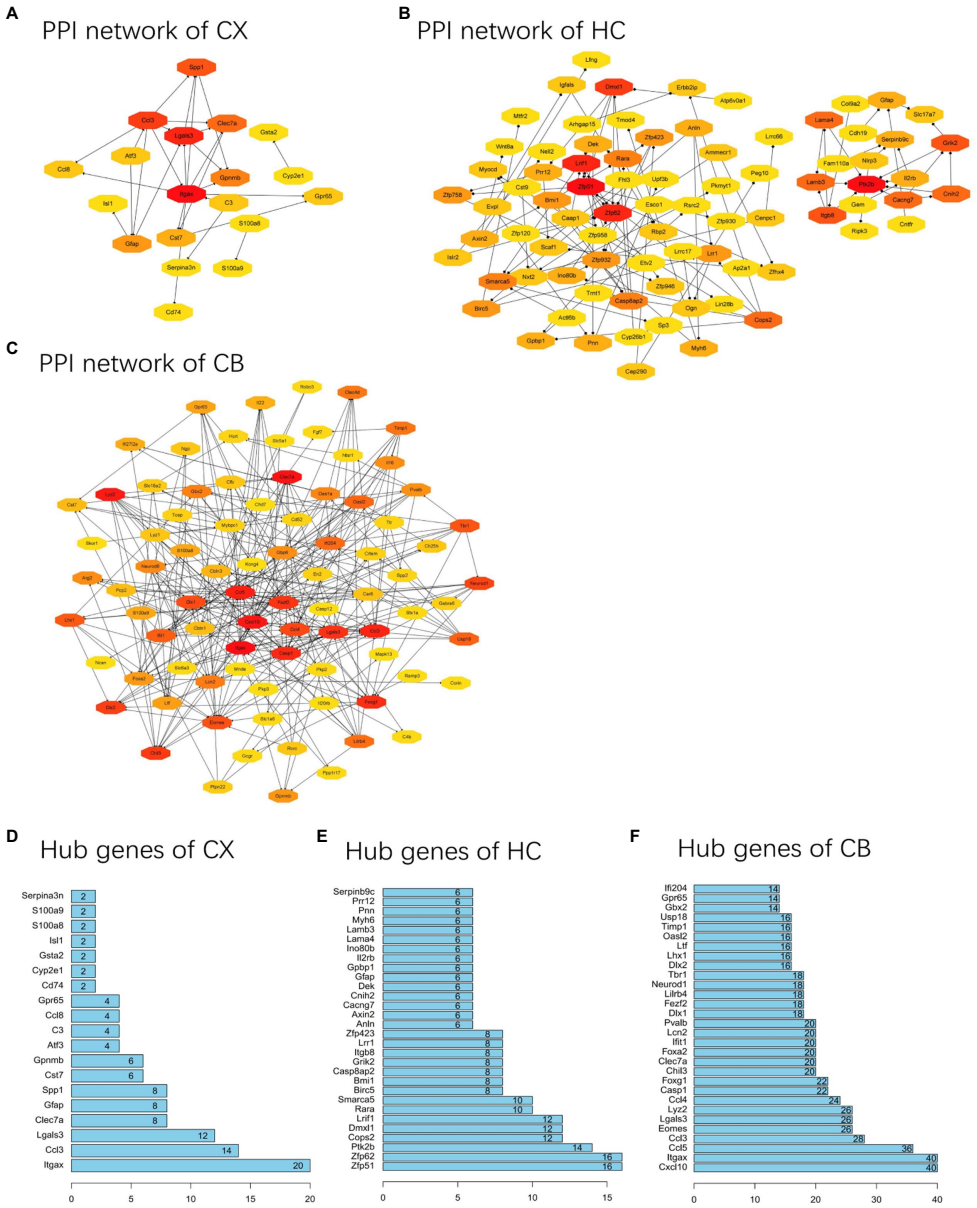
PPI networks and hub genes of DEGs. **(A-C)** The results of network analysis based on Cytoscape. The depth of color represents the degree of gene in network analysis. **(D-F)** The results of network analysis based on R software. The x-axis represents the number of gene connections in network. CX, cerebral cortex; HC, hippocampus; CB, cerebellum.

PPI networks were constructed using STRING and analyzed using RGui and Cytoscape. The cortical PPI network consisted of 19 nodes and 56 edges with an average node degree of 3.47 and an average clustering coefficient of 0.51. The hippocampal PPI network consisted of 121 nodes and 242 edges with an average node degree of 2.38 and an average clustering coefficient of 0.09. The cerebellar PPI network consisted of 94 nodes and 500 edges with an average node degree of 5.83 and an average clustering coefficient of 0.44. In the Cytoscape analysis, we obtained the hub genes depending on the degree of gene expression, and the results were visualized using Cytoscape. The network diagrams list only one-way edge results. Itgax is a hub gene involved in cerebral cortical aging (Figure 7A). Zfp52 and Zfp62 were hub genes involved in hippocampal aging (Figure 7B). Itgax and Cxcl10 were hub genes involved in cerebellar aging (Figure 7C). These four genes were all upregulated in the DEGs. The degrees of hub genes were as follows: Itgax: 20 (in the cortical network)/40 (in the cerebellar network); Zfp51 and Zfp62: 16; Cxcl10: 40. In RGui analysis, we obtained the same results for hub gene identification. The top 30 genes of each brain region are presented in a bar plot based on the number of gene connections in the PPI network (Figures 7D–F). The functional enrichment items and pathways that contain the hub genes or co-DEGs (S100a8) are also listed in Tables 2–4 (in which only the top three items according to *p*-values are listed). These results suggest that different brain regions may undergo different molecular changes during aging (Table 5).

**Table 3:** GO and KEGG items.

Gene symbol	ID of GO/KEGG term	Description	GeneRatio	*p*-value
Itgax	GO:0005178	Integrin binding	4/28	1.61E-05
	GO:0050839	Cell adhesion molecule binding	4/28	0.00023
	mmu05152	Tuberculosis	4/18	0.00038
	mmu04610	Complement and coagulation cascades	2/18	0.01467
S100a8	GO:0030593	Neutrophil chemotaxis	7/29	8.87E-12
	GO:0071621	Granulocyte chemotaxis	7/29	4.16E-11
	GO:1990266	Neutrophil migration	7/29	4.64E-11
	mmu04657	IL-17 signaling pathway	2/18	0.01467

The top three items containing hub genes or S100a8 of CX according to *p*-values are listed if there are more than three items.

**Table 4:** GO and KEGG items.

Gene symbol	ID of GO/KEGG term	Description	GeneRatio	*p*-value
Zfp51	mmu05168	Herpes simplex virus 1 infection	13/99	0.00156
S100a8	GO:0006919	Activation of cysteine-type endopeptidase activity involved in apoptotic process	5/263	0.00075
	GO:0052547	Regulation of peptidase activity	11/263	0.00268
	GO:0030595	Regulation of cysteine-type endopeptidase activity involved in apoptotic process	7/263	0.00033

The top three items containing hub genes or S100a8 of HC according to *p*-values are listed if there are more than three items.

**Table 5:** GO and KEGG items.

Gene symbol	ID of GO/KEGG term	Description	GeneRatio	*p*-value
Itgax	mmu04610	Complement and coagulation cascades	3/62	0.02671
Cxcl10	GO:0051924	Regulation of calcium ion transport	11/126	5.83E-08
	GO:0030595	Leukocyte chemotaxis	10/126	6.83E-08
	GO:0050900	Leukocyte migration	12/126	1.18E-07
	mmu04657	IL-17 signaling pathway	5/62	0.00043
	mmu04061	Viral protein interaction with cytokine and cytokine receptor	5/62	0.00047
	mmu04620	Toll-like receptor signaling pathway	5/62	0.00060
S100a8	GO:0030595	Neutrophil chemotaxis	10/126	6.83E-08
	GO:0050900	Leukocyte migration	12/126	1.18E-07
	GO:0030593	Neutrophil chemotaxis	7/126	3.62E-07
	mmu04657	IL-17 signaling pathway	5/62	0.00043

The top three items containing hub genes or S100a8 of CB according to *p*-values are listed if there are more than three items.

### Potential gene–miRNA interactions

The miRBD, TargetScan, miRWALK, and DIANA tools were used to identify the top three miRNAs targeting each hub gene and co-DEGs (S100a8) based on the predictive scores. The predicted miRNAs for Itgax are mmu-miR-185-3p, mmu-miR-7,048-5p, and mmu-miR-379-5p. The predicted miRNAs for Zfp51 are mmu-miR-23b-3p and mmu-miR-7,655-3p. The predicted miRNAs for Zfp62 are mmu-miR-181a-5p, mmu-miR-181b-5p, and mmu-miR-181c-5p. The predicted miRNAs for Cxcl10 are mmu-miR-101b-3p, mmu-miR-101a-3p, and mmu-miR-126b-5p (Table 6). These results implicate miRNAs in aging in different brain regions.

**Table 6:** Interaction between hub genes and miRNA.

Brain region	Gene symbol	Predicted miRNA
CX	Itgax	mmu-miR-185-3p, mmu-miR-7,048-5p, mmu-miR-379-5p
HC	Zfp51	mmu-miR-23b-3p, mmu-miR-7,655-3p
	Zfp62	mmu-miR-181a-5p, mmu-miR-181b-5p, mmu-miR-181c-5p
CB	Cxcl10	mmu-miR-101b-3p, mmu-miR-101a-3p, mmu-miR-126b-5p

### Relationship between aged-related nervous system disease and hub genes/S100a8

**Figure 8:**
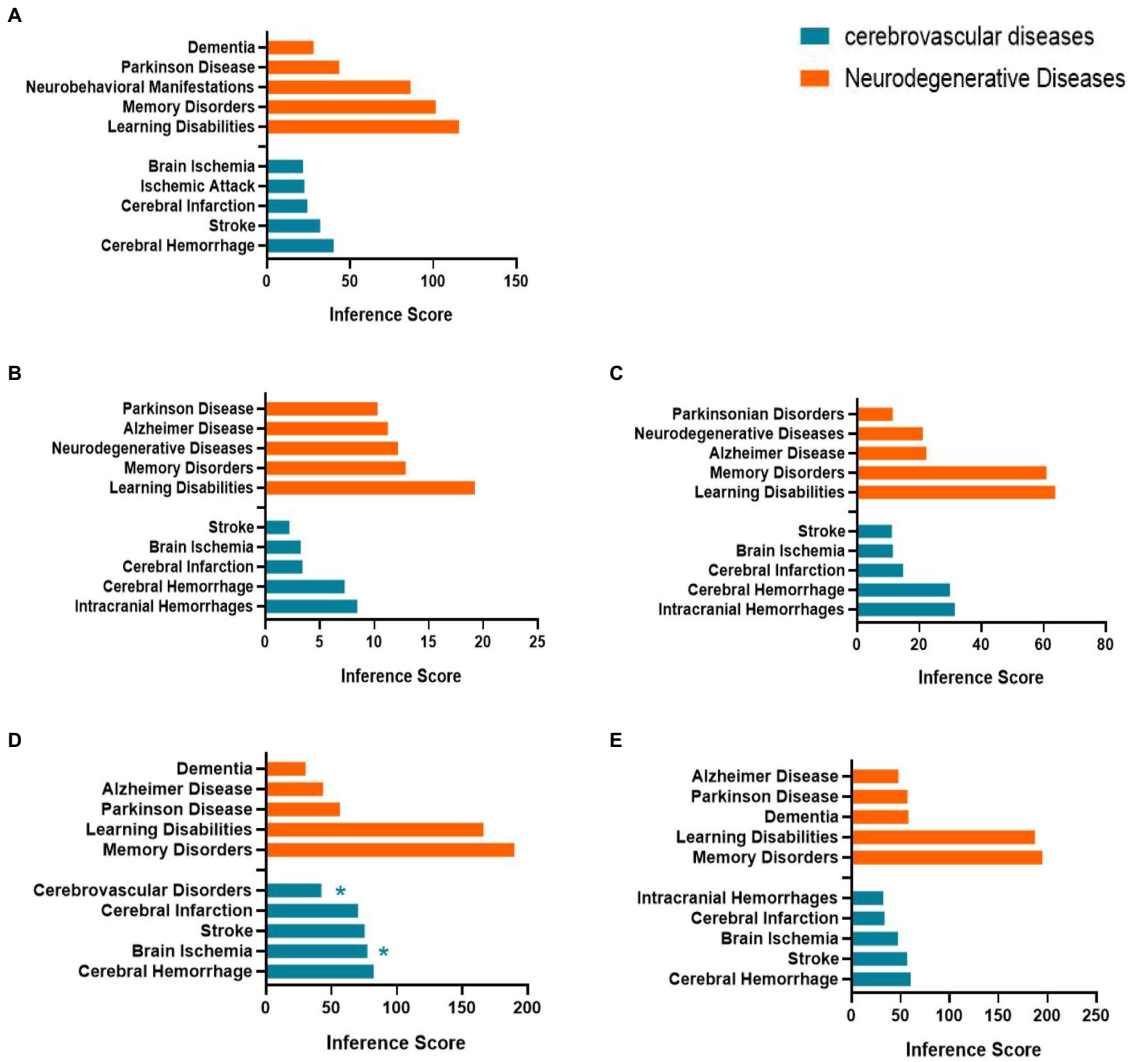
Relationship between Itgax **(A)**, Zfp51 **(B)**, Zfp62 **(C)**, Cxcl10 **(D)**, S100a8 **(E)** and aged related nervous system diseases. Data are from the Comparative Toxicogenomics Database. The top five of neurodegenerative diseases and age-related cerebrovascular diseases of each hub gene and S100a8 are listed in the bar charts based on the inference score. *, Direct evidence of marker or molecular change in this disease.

We used the CTD database to explore the relationship between age**-**related nervous system diseases and hub genes/co-DEGs (S100a8) based on the inference scores. The neurodegenerative diseases for the hub genes and S100a8 included dementia, Parkinson’s disease, neurobehavioral abnormalities, memory disorders, learning disabilities, and Alzheimer’s disease. The age**-**related cerebrovascular diseases for the hub genes and S100a8 included cerebrovascular disorders, stroke, brain ischemia, ischemic attack, cerebral infarction, cerebral hemorrhage, and intracranial hemorrhage (Figure 8).

### Expression level and regional specificity of hub genes and S100a8

**Figure 9:**
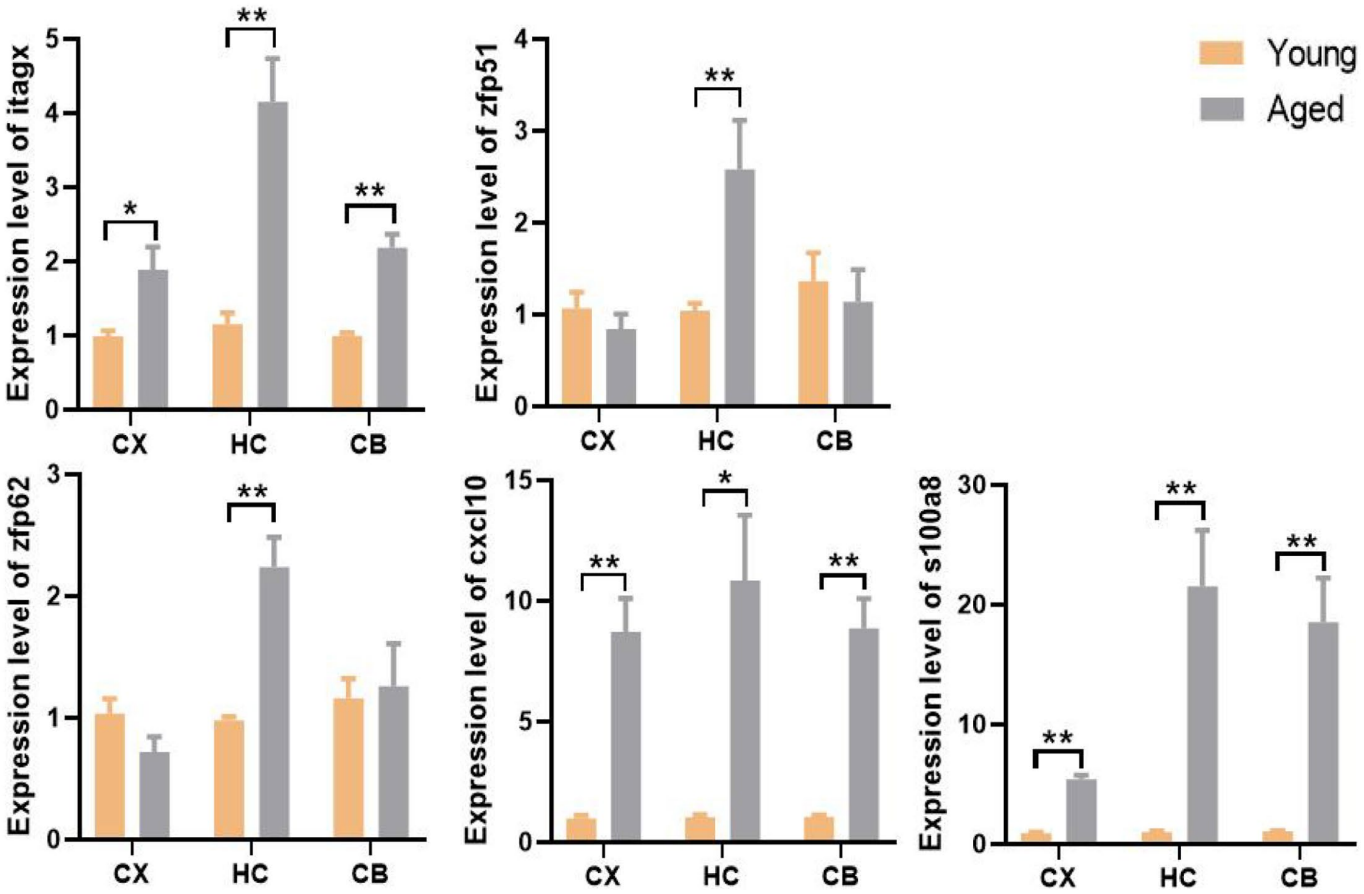
The expression level and regional specificity of the hub genes and S100a8 in mice (Measured by RT-qPCR method). Sample size (Young and aged samples account for half of each panel): Itgax (CX: *n* = 32, HC: *n* = 14. CB: *n* = 32); Zfp51 (CX: *n* = 14, HC: *n* = 32. CB: *n* = 14); Zfp62 (CX: *n* = 14, HC: *n* = 32. CB: *n* = 14); Cxcl10 (CX: *n* = 14, HC: *n* = 14. CB: *n* = 32); S100a8 (CX: *n* = 32, HC: *n* = 32. CB: *n* = 32); *p*-values of Itgax of CX, Zfp51 of HC, S100a8 of HC and CB are calculated with Wilcoxon rank-sum test. *p*-value of other panel are calculated with t -test. Results are expressed as mean ± SEM. CX, cerebral cortex; HC, hippocampus; CB, cerebellum. Y, young; A, aged; ^*^p < 0.05; ^**^p < 0.01.

We used the qPCR method to validate gene expression levels. Consistent with the results of the bioinformatic analysis, the levels of Itgax, Zfp51, Zfp62, Cxcl10, and S100a8 were all significantly upregulated in the corresponding region of aged samples. Surprisingly, only the upregulation of Zfp51 and Zfp62 was restricted to the HC, whereas Itgax and Cxcl10 were significantly upregulated in all three regions in aged samples, similar to the co-DEGs (S100a8; Figure 9). This evidence further corroborates the level change of these genes in the aging of different brain regions and indicates the fundamental role of neuroinflammation in brain aging.

## Discussion

The study findings reveal that the molecular changes and key genes involved in aging exhibit regional differences in the brain. Most of the changes seem to be closely related to neuroinflammation. Although several previous studies based on bioinformatics analyses have already focused on normal brain aging, none have explored the key genes of aging depending on the brain region and multiple online datasets (Li et al., 2018; Xu et al., 2022). Whether there are regional differences in molecular changes in the aging brain is unclear. Therefore, this study fills this gap to a certain extent.

GO terms of cerebral cortical aging strongly suggest that leukocyte (especially neutrophils) chemotaxis and migration is an important change in cortical aging, and integrins (αβ-heterodimer CAMs), which are exclusively expressed on the surface of all leukocytes, play a key role in cerebral cortical aging (Wen et al., 2022). Previous studies have shown that aging increases blood–brain barrier permeability, which facilitates neutrophil invasion in the aging brain (Roy-O'Reilly et al., 2020). Integrin activation contributes to the slow rolling, adhesion, and recruitment of leukocytes during this invasion process, thus maintaining immune responses and inflammation levels (Sun et al., 2021). Chemokines are chemotactic agonists that regulate integrin activation through GPCRs, such as monocyte C-C motif chemokine receptor 2 (CCR2; Sun et al., 2021). Thus, the CCR may also be involved in leukocyte recruitment in aging CX. This is supported by evidence that GPCRs and CCR receptor levels are also altered in CX from aged rodents (Cartier et al., 2005; Gu et al., 2021). In addition, what can support the suggestion mentioned earlier is that the DEGs of cortical tissue were enriched in several inflammatory signaling pathways in the KEGG results. Coincidentally, integrin alpha x (Itgax) or CD11c (the latter has been already used in numerous studies) that encodes an integrin family protein was the hub gene of cerebral cortical aging in our results. Several studies have shown that the level of Itgax increases with age and is related to CX neuroinflammation (Hart et al., 2012; Raj et al., 2017; Kang et al., 2018; Sato-Hashimoto et al., 2019). In addition to its role in normal aging, Itgax participates in the pathological processes of neurodegenerative diseases such as AD (Rangaraju et al., 2018; Benmamar-Badel et al., 2020). Combined with this evidence, our results suggest that Itgax-mediated neutrophil invasion plays a key role in cortical aging. However, despite inducing neuroinflammation, upregulation of Itgax enhances cellular phagocytosis and thus promotes the clearance of Aβ plaques, which is beneficial for the brain (Benmamar-Badel et al., 2020). Therefore, whether the increase in Itgax is an important factor in aging CX is unclear and requires further study.

The enrichment results for hippocampal aging appear more complex, but the abnormality of neurotransmitter transmission (especially excitatory neurotransmitters) seems to play a critical role. A decline in the level of excitatory neurotransmitters, such as glutamate, is a robust marker of brain aging (Roalf et al., 2020). Neurotransmitter transmission is regulated by calcium-dependent exocytosis (Wu et al., 2014). Actin cytoskeleton remodeling precisely controls exocytosis by actin filaments, thus ensuring normal granule transport in the synaptic cleft (Bader et al., 2004). Abnormal neurotransmission, oxidation of calcium channels, decrease in exocytosis, and levels of actin protein (for example, drebrin) have all been found in aging HC in previous studies (Lalo et al., 2014; Patel and Sesti, 2016; Willmes et al., 2017; Roalf et al., 2020; Tao et al., 2021). These alterations lead to impairment of long-term potentiation, which is directly responsible for cognitive decline (Kojima and Shirao, 2007; Ivanov et al., 2009; Jung et al., 2015). In addition, the enrichment results may also emphasize that neuroinflammation induced by microglia (a type of resident myeloid cells in the brain) and apoptosis are involved in hippocampal aging. Zfp51 and Zfp62 were identified as hub genes involved in hippocampal aging in our study. These two genes are zinc finger encoding genes and have the same evolutionarily conserved C2H2-link sequence. However, these two genes have not been thoroughly investigated. To the best of our knowledge, no study has focused on the relationship between these two genes and brain aging. Therefore, research on the role of these two genes in hippocampal aging is required.

CB appears to have a relatively slow rate of aging. The role of CB in cognitive decline is enigmatic. Therefore, it is not surprising that CB has been dismissed or even ignored in studies on brain aging. However, some recent studies have shown that pathological changes in CB may also be associated with neurodegenerative disorders, highlighting the significance of CB in normal aging (Liang and Carlson, 2020). Most GO items associated with cerebellar aging are related to neuronal development and differentiation. This suggests that impaired neurogenesis plays a fundamental role in cerebellar aging. As a compensatory mechanism for neuronal death, neurogenesis can restore the brain to a more youthful state during aging. However, neurogenesis in the CB is significantly impaired with age (Elkholy and Al-Gholam, 2018; Isaev et al., 2019). In addition, DEGs enriched for calcium ion transport provide further confirmation for this suggestion because calcium transport is indispensable for cell proliferation and neural differentiation in neurogenesis (Toth et al., 2016). However, during aging, the number of calcium channels necessary for transport decreases (Martini et al., 1994; Chung et al., 2001). A series of functions and pathways related to leukocyte reaction and inflammatory cytokine responses are consistent with the results that Itgax and Cxcl10 were the hub genes of cerebellar aging because these two genes are closely related to the inflammatory reaction. In addition to leukocyte-induced inflammation, Itgax is related to neurogenesis (Blau et al., 2012). Similar to Itgax, Cxcl10, a chemokine superfamily coding protein, is expressed in astrocytes, glial cells, dendritic cells, and neutrophils, and is closely related to neuroinflammation (Fazia et al., 2020). Cxcl10 is upregulated in aged brains (Hasegawa-Ishii et al., 2016; Palomera-Ávalos et al., 2017; Tennakoon et al., 2017; Hu et al., 2019). Expression of Cxcl10 can induce the release of the senescence-associated secretory phenotype, which consists of multiple inflammatory factors and promotes brain aging (Cardoso et al., 2018). The upregulation of inflammatory cytokines is usually accompanied by impaired neurogenesis (Kim et al., 2016). This evidence suggests that Itgax and Cxcl10-mediated neuroinflammation may be the driving force for the impairment of neurogenesis in CB and thus responsible for cerebellar aging.

Subsequently, we identified that S100a8 was the only co-DEG of all three brain regions. A previous study showed that the level of S100A8, the eponymous protein encoded by the S100a8 gene, is increased in the prefrontal cortex, hippocampus, and cerebellum of aged rats and plays a critical role in inflammatory regulation (Hamasaki et al., 2019). Similar results were obtained in aged mice and a mouse model of AD (Lodeiro et al., 2017). As the brain ages, some brain regions contain high levels of S100 proteins. Among these proteins, S100A8 has the highest staining intensity (Hoyaux et al., 2000). Most S100A8 proteins are aggregated around amyloid plaques neighboring activated microglia, which form a positive feedback loop between S100A8 and Aβproduction (Lodeiro et al., 2017). This may explain why amyloid deposition and neuroinflammation are common phenomena in the aging brain (Singh-Bains et al., 2019; Zhang et al., 2021). After gene identification, we were interested in determining whether the upregulation of hub genes had regional specificity. Therefore, we measured the level of each hub gene in each brain region. We were surprised to find that only the upregulation of Zfp51 and Zfp62 was restricted to the HC, whereas Itgax and Cxcl10 were significantly upregulated in all three regions of the aged brain, similar to S100a8. This suggests that Zfp51 and Zfp62 may be specific biomarkers of hippocampal aging, and neuroinflammation may be a common and basic mechanism of brain aging because the expression of Itgax, Cxcl10, and S100a8 are all strongly related to inflammatory reactions, as mentioned above.

Considering that the hub genes are also regulated by miRNA, we predicted the top three selected miRNAs that targeted each hub gene and S100a8 based on the predictive scores. Supporting evidence is also found for these predictions. In one study, miR-185-3p was related to aging and frailty in exosomes derived from plasma (Ipson et al., 2018). Jiang and colleagues described a decreased level of miR-23b-3p in HC from aged mice (Jiang et al., 2022). The authors also reported that upregulation of this miRNA attenuates cognition impairment. In another study, upregulation of miR-181a-5p was demonstrated in HC of AD mice (Rodriguez-Ortiz et al., 2020). The authors described that this upregulation negatively modulates synaptic plasticity, leading to memory impairment. Angiotensin II induces brain vascular smooth muscle cell (BVSMC) senescence by negatively regulating miR-181b-5p (Li et al., 2019; Bai et al., 2021). Therefore, the downregulation of this miRNA in HC may be responsible for the aging of BVSMC. Microarray analysis has shown that miR-181c-5p is associated with hippocampal aging (Zhou et al., 2016). MiR-101b-3p controls neuronal plasticity, indicating a role in the impairment of neurogenesis in cerebellar aging (Codocedo and Inestrosa, 2016). This evidence functionally implicates miRNAs in the aging brain. However, their relationship to hub genes and their effects on the aging of specific brain regions need to be confirmed. These four hub genes and S100a8 are all associated with the most common age-related nervous system diseases, and this further suggests that changes in the levels of these genes may play a critical role in cognitive decline and neurovascular aging; however, more animal and clinical studies are needed to confirm the biological role of these genes in normal brain aging.

This study has some limitations. First, this was a small sample study from the point of both bioinformatics analysis and animal experiments. In contrast, we only explored the potential mechanism at the gene expression level, but gene expression may not be directly equivalent to protein expression and biological effects. Therefore, more animal and clinical studies are needed, other comprehensive databases of biometric information should be applied to bioinformatics analysis, and the results should be validated through different experimental methods.

## Conclusion

This study revealed that the molecular signatures of aging exhibit regional differences in the brain and seem to be closely related to neuroinflammation. Integrin-mediated neutrophil invasion, abnormal synaptic transmission, and impaired neurogenesis due to inflammation may play key roles in the aging of CX, HC, and CB. Itgax, Zfp51, Zfp62, Cxcl10, and S100a8 are potential targets for preventing aging in the brain. Further studies are required to confirm these results.

## Data availability statement

The original contributions presented in the study are included in the article/Supplementary material, further inquiries can be directed to the corresponding author.

## Ethics statement

The animal study was approved by the Animal Care Welfare Committee of Guangxi Medical University.

## Author contributions

XS and LX were responsible for the data acquisition, analysis, experimental operation, literature search, and writing of the manuscript. JL, XT, and BL curated the data and created the figures. MC designed the study and revised the manuscript. All authors contributed to the article and approved the submitted version.

## Funding

This study was supported by the National Natural Science Foundation of China (grant numbers: 81860333, 82072128, and 82160372). The funders had no role in the study design, data collection and analysis, decision to publish, or manuscript preparation.

## Conflict of interest

The authors declare that the research was conducted in the absence of any commercial or financial relationships that could be construed as potential conflicts of interest.

## Publisher’s note

All claims expressed in this article are solely those of the authors and do not necessarily represent those of their affiliated organizations, or those of the publisher, the editors and the reviewers. Any product that may be evaluated in this article, or claim that may be made by its manufacturer, is not guaranteed or endorsed by the publisher.

## Footnotes

^1^ http://www.ncbi.nlm.nih.gov/geo

^2^ http://www.sangerbox.com

^3^ http://bioinformatics.psb.ugent.be/webtools/Venn/

^4^ https://metascape.org/gp/index.html

^5^ https://david.ncifcrf.gov/tools.jsp

^6^ https://cn.string-db.org

^7^ https://cytoscape.org/

^8^ https://mirdb.org/

^9^ https://www.targetscan.org

^10^ http://mirwalk.umm.uni-heidelberg.de/

^11^ http://diana.imis.athena-innovation.gr/DianaTools/

^12^ http://ctdbase.org/
